# What Do Patients Think About Their Radiation Oncologists? An Assessment of Online Patient Reviews on Healthgrades

**DOI:** 10.7759/cureus.2165

**Published:** 2018-02-06

**Authors:** Arpan V Prabhu, Simrath Randhawa, David Clump, Dwight E Heron, Sushil Beriwal

**Affiliations:** 1 Department of Radiation Oncology, University of Pittsburgh Cancer Institute, UPMC

**Keywords:** healthgrades, online reputation, internet, digital identity, radiation oncology

## Abstract

Introduction

An increasing number of patients search for their physicians online. Many hospital systems utilize Press-Ganey studies as internal tools to analyze patient satisfaction, but independent third-party websites have a large presence online. Patients’ trust in these third-party sites may occur despite a low number of reviews and a lack of validity of patients’ entries. Healthgrades.com has been shown as the most popular site to appear on Google searches for radiation oncologists (ROs) in the United States (US). The aim of this study was to analyze patient satisfaction scores and the factors that influence those scores for American ROs on Healthgrades.

Methods

The physician ratings website Healthgrades was manually queried to obtain reviews from all Medicare-participating ROs with reviews (*n*=2,679). Patient Review Satisfaction Scores (PRSS) were recorded in response to a variety of questions. All information in the survey was scored from 1 (poor) to 5 (excellent) for the following characteristics: likelihood to recommend (LTR), office environment, ease of scheduling, trust in the physician’s decision, staff friendliness, ability of the physician to listen and answer questions, ability of the physician to explain the condition, and whether the physician spent sufficient time with the patients. Associations amongst these factors were considered by computing Spearman correlation coefficients and utilizing Mann-Whitney and Kruskal-Wallis tests.

Results

The ROs’ mean LTR score was 4.51±0.9 (median 5.0, 66% received the highest possible score of 5; 95% received a score>2). Patient reviews per RO ranged from 1 to 242 (4.50±0.9, median 2.0). LTR scores correlated very strongly with physician-related factors, ranging from *r*=0.85 (with appropriate time spent with patients) to *r*=0.89 (with level of trust in physician). LTR scores were not statistically significantly associated with gender, wait time, ROs’ years since graduation, academic status, or geographic region.

Conclusion

Satisfaction scores for ROs on a leading physician ratings website are very strong, and most patients leaving reviews are likely to recommend their own ROs to their friends and family. Understanding online ratings and identifying factors associated with positive ratings are important for both patients and ROs due to the recent growth in physician-rating third-party sites. ROs should have increased awareness regarding sites like Healthgrades and their online reputation.

## Introduction

Over the past decade the percent of people using the Internet to obtain health information has risen from 20% to 60% [1]. In addition to researching articles about health conditions and treatments, patients can access information about their physicians. Although many hospital systems utilize Press-Ganey studies as internal tools to analyze patient satisfaction, independent third-party websites have a large and noticeable presence online.

More than 90% of physicians have professional information available online that are often on these third-party physician rating websites [2]. Such physician-rating websites function to provide free basic information about physicians’ backgrounds while additionally enabling users to enter reviews for specific physicians [3]. Patients’ trust in these third-party sites may occur despite a low number of reviews and a lack of validity of patients’ entries, as previous work has shown that 75% of patients who frequent these third-party sites are influenced when choosing a physician [4]. In fact there has been work to suggest that comments from such sites are actually more persuasive on consumers than statistically summarized data from much larger, representative samples of patients [5].

Healthgrades.com has been shown to be the most popular physician ratings site with the highest frequency on Google searches for radiation oncologists (ROs) in the United States (US) [6]. On Healthgrades and other third-party sites, patients can score physicians for a variety of factors as well as give an overall satisfaction score, collectively referred to as patient review satisfaction scores (PRSS) [3]. Previous studies have evaluated PRSS across various specialties, including radiology and interventional radiology [7-8]. Prior work investigating PRSS in the field of radiation oncology (RO), however, has been limited by small sample sizes and brief surveys [9].

This study aims to evaluate PRSS and the factors that influence those scores for ROs across the United States on Healthgrades. The goal is to better understand online physician ratings for ROs and improve ROs’ awareness of their online reputations.

## Materials and methods

This study did not require IRB approval because it utilized publicly available federal databases and web-accessible data sources. The methodology to acquire the study population has been previously described by Prabhu et al. [6]; the remaining methodology for this paper follows that presented by Obele et al. [8] and is expanded below.

Study population

The Centers for Medicare and Medicaid Services (CMS) Physician Compare National Downloadable File (PCNDF) was used to generate a list of radiation oncologists [10]. The data were accessed and de-duplicated using National Provider Identifier (NPI) numbers on September 23, 2016. All remaining entries were included for analysis (*n*=4,444). The PCNDF captures all physicians enrolled in Medicare fee-for-service, or about 91% of the physicians in the United States [11] and is comprehensive and representative of US physicians.

The PCNDF list of all radiation oncologists was downloaded as a .csv file and analyzed using Python (version 2.7) and Pandas, an open-source library for working with data in Python [12]. Information on first name, last name, NPI number, gender, degree type (MD or DO), medical school graduation year, and practice location city and state was extracted from the PCNDF dataset. The April 2017 Association of Residents in Radiation Oncology (ARRO) Directory [13] and the departmental websites of academic programs were used to compile an external database listing academic radiation oncologists in the United States. Departmental websites were accessed in June and July 2017. This was then consulted to verify the academic status of physicians in this study.

Data collection

Healthgrades was the physician rating website used to obtain patient reviews in this analysis. Each radiation oncologist with at least one patient rating on the Healthgrades search tool was included in this study (n=2,679). Demographic data is presented in Table 1. All ratings were scaled from 1 to 5 (1=poor, 2=fair, 3=good, 4=very good, and 5=excellent). The global measure of patient satisfaction in all reviews was found by the “likelihood to recommend to family and friends” (LTR) rating. Other descriptive factors were classified as office-related factors or physician-related factors. Office-related factors included “ease of scheduling appointments,” “office environment in terms of cleanliness and comfort,” and “staff friendliness and courteousness.” Physician-related factors consisted of “how well the provider explains the medical condition,” “level of trust in the provider,” “how well the provider answers questions,” and “did the provider spend appropriate amount of time with patients.” Additionally, patients also indicated total wait time using a scale of 1-5 (1= less than 10 minutes, 2= 10-15 minutes, 3= 16-30 minutes, 4= 31-45 minutes, 5= >45 minutes). Geography was classified under the following scale: 1=Northeast, 2=Southeast, 3=Midwest, 4=West. States were classified in an appropriate region following designations given by the US Census Bureau [14]. Data was additionally sorted by gender with male ROs receiving a score of 1 and females a score of 2. Finally, for classifying academic status, non-academics were given a score of 1 while academics were given a score of 2.

**Table 1:** Summary of all 2,679 Medicare-participating self-designated radiation oncologists with patient ratings available on Healthgrades *Data unavailable for *n*=8

Gender	n (%) or mean ± standard deviation
Male	2008 (75.6)
Female	671 (24.4)
Years Since Graduation*	25.3 ± 4.7 (median 25, range 1-53)
0-10 years	11.8%(315)
11-20 years	24.7%(664)
21-30 years	29.2%(783)
31+ years	33.9%(909)
Academic Status	
Academic	554 (20.6)
Non-academic	2125 (79.4)
Geographic region	
West	612 (22.7)
East	610 (22.7)
Southeast	844 (31.4)
Northeast	613 (22.8)
Number of patient reviews	4.50± 0.9 (median 2, range 1-242)

Data analysis

Summary Statistics

Responses were summarized by calculating standard summary statistics. 

Mean LTR Correlation with Various Factors

Spearman correlation coefficients were computed with a value between 0.81 and 1.00 indicating very strong agreement, 0.61 and 0.80 indicating strong agreement, and lower than 0.61 not being considered a significant agreement. Correlation coefficients were calculated between LTR and all office-related factor scores, physician-related factor scores, waiting times, years of experience, and number of patient reviews.

Mann-Whitney Test

Mean LTR scores were computed for each gender and compared through the Mann-Whitney Test. Mean LTR scores were also compared between academic and non-academic physicians as well as amongst physicians with varying time since graduation. Analysis was conducted amongst the specific subset of patients with reported waiting times of <15 minutes and >30 minutes by calculating each mean LTR value and comparing them using the Mann Whitney test. Additionally, the subset of physicians with 10 or more reviews was compared to the subset of physicians with less than 10 reviews by calculating each mean LTR value and using the Mann Whitney test for comparison.

Mean LTR, physician-factor, and office-factor scores for the subset of 741 patients who gave a score >0.5 in magnitude less than the highest LTR possible were also computed. The decision to use 4.5 as the cutoff for creating two subsets of data with different LTR scores for comparison was an arbitrary threshold we established for simplicity. The Mann Whitney test was used to specifically compare the mean LTR score between this 741 patient subset and the sample of all 2,679 patients.

Kruskal Wallis Test

Mean LTR score was determined for each region, and the Kruskal-Wallis test was used to compare the mean between each region. 

Internal Validity

Cronbach’s alpha statistic was computed amongst individual office factors and physician factors to test how well these factors were able to assess the same underlying construct.

## Results

Data collection and summary statistics

**Figure 1:**
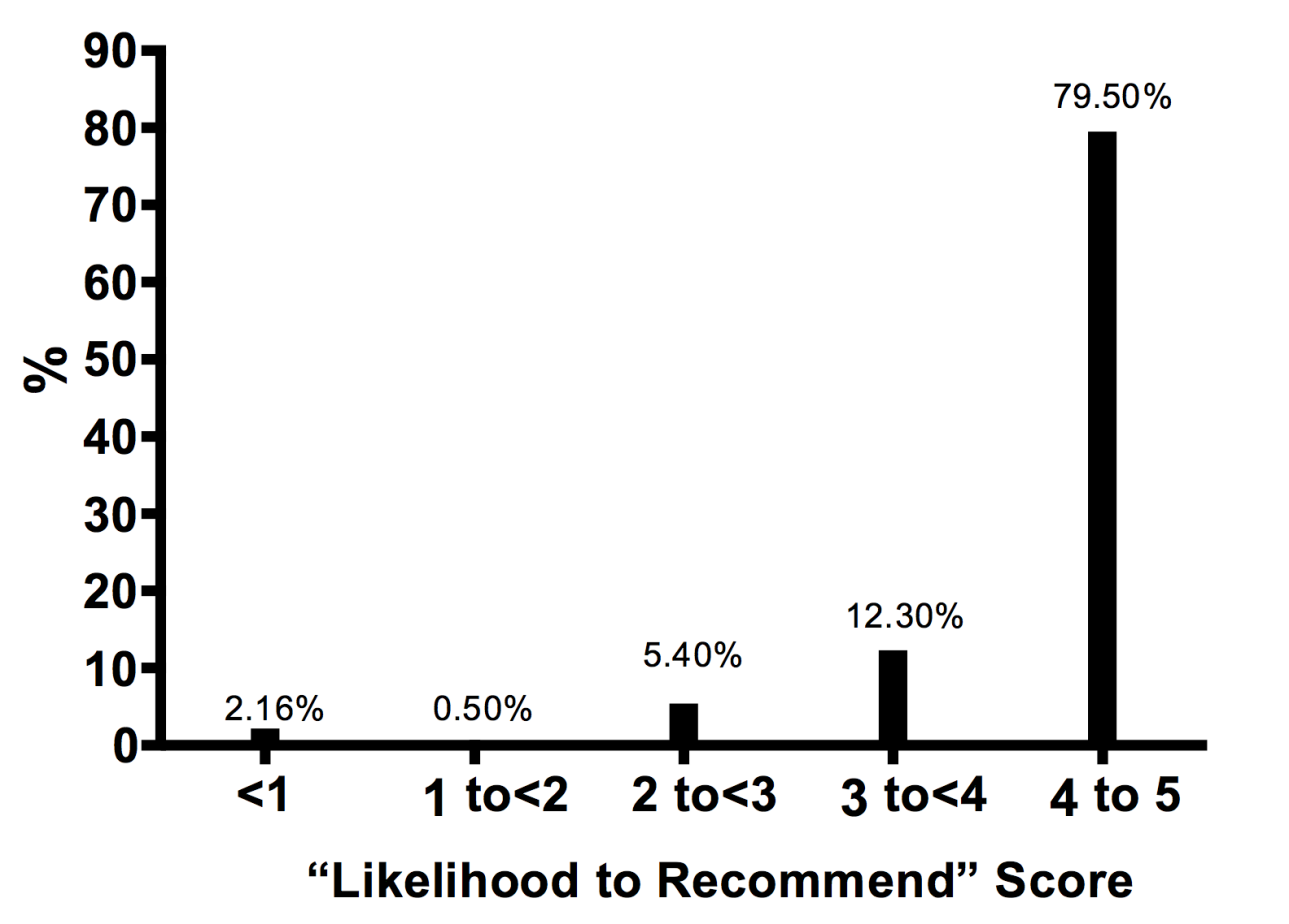
Distribution of overall likelihood-to-recommend score among included radiation oncologists

A summary of all 2,679 Medicare-participating ROs with patient ratings available through the Healthgrades search engine is provided (Table 1). The number of patient reviews per RO ranged from 1 to 242 (4.50±0.9, median 2.0). Exactly 215 of 2679 physicians (8.0%) received over 10 reviews. The mean scores for survey items and their correlation with the LTR score can be viewed (Table 2). The most common wait time was less than 10 minutes (50%). Only 3% of patients’ waiting time exceeded 30 minutes. The mean LTR score ranged from very good to excellent (4.51±0.9, median 5.0) (Figure 1), and 1791 of 2679 ROs (67%) received the highest possible LTR score. About 97% (2565/2679) of ROs received a score of good or higher. 

**Table 2:** Mean score for survey items and correlations with likelihood-to-recommend (LTR) score Significant correlations are in bold text *Gender was assigned on a scale of Male=1 Female=2 Graduation year was assigned as follows: <10 years ago=1. 10-20 years ago=2. 21-30 years ago=3. 31+ years ago=4 Academic status was assigned as the following: Nonacademic: 1 Academic: 2 Location assigned as following: Northeast: 1 Southeast: 2 Midwest: 3 West: 4

	n	Mean	Correlation with LTR
LTR	2679	4.51±0.8	n/a
Office-related factors			
Ease of scheduling appointments	2671	4.72±0.4	0.59
Office environment	2671	4.78±0.7	0.53
Staff friendliness	2671	4.70±0.7	0.52
Physician-related factors			
Spends appropriate amount of time with patient	2674	4.64±0.8	0.86
How well provider explains medical condition	2676	4.65±1.6	0.89
How well provider listens and answers questions	2676	4.64±0.8	0.89
Level of trust in provider’s decision	2679	4.68±1.0	0.89
Number of patient reviews:	2679	4.50±0.87	0.02
Gender^*^	2679	1.25±1.0	0.02
Graduation year^*^	2676	2.85±0.87	0.02
Academic status^*^	566	1.17±0.51	0.01
Location^*^	2674	2.44±0.81	n/a

Mean LTR Correlation with Variables

The LTR score was strongly correlated with the ability of a provider to explain medical conditions (*r*=0.887), how well they provided answers to patient questions (*r*=0.889), and whether the patient determined if the RO spent appropriate time with them (*r*=0.858). No significant correlation was found between LTR and ease of scheduling appointments (*r*=0.586), staff friendliness (*r*=0.521), the patient’s perception of the office environment (*r*=0.533), and waiting time (*r*=0.224). LTR showed no correlation with number of reviews (*r*=0.02) or academic/non academic level status (*r*=0.01).

Mann-Whitney Test

The LTR score showed no association with ROs’ years since graduation (*p*=0.81), academic status (*p*=0.76), or gender (*p*=0.92). No significant difference was found between mean LTR (*p*=0.14) for patients who waited under 15 minutes (4.56±0.8) and those who waited over 30 minutes (4.38±1.0).

Amongst the subset of 741 ROs who received an LTR<4.5, statistically significant differences were found for the mean LTR score between physicians who received good or higher scores in staff friendliness (*p*=0.01), time well spent (*p*=0.01), office environment (*p*<0.01), ability to explain conditions (*p*<0.01), and trustworthiness (*p*=0.01). Scores less than very good (<4 out of 5) were given for physician characteristics at three times the frequency of office-related factors.

There was a significant difference between the mean LTR among ROs with 10 or more reviews and fewer than 10 reviews (*p*=0.01). No significant difference was found for time well spent (*p*=0.15), ability to answer questions (*p*=0.13), ability to explain conditions (*p*=0.12), and trustworthiness (*p*=0.10).

Kruskal Wallis Test

The mean LTR score showed no statistically significant association (*p*=0.08) with any geographical region (Northeast 4.54±0.8, Southeast 4.56±0.8, Midwest 4.50±0.9, West 4.48±0.8).

Internal Validity

Internal validity was assessed by computing Cronbach’s α value among the individual office factors (α =0.981) and the individual physician factors (α =0.851). These values indicated the survey items adequately assessed the underlying construct and were devoid of interfering confounding variables.

## Discussion

By identifying all Medicare-designated ROs and manually searching patient-generated data from Healthgrades, a popular physician ratings website, we found ROs to have very good to excellent patient satisfaction ratings. About 91% of patients were found likely to recommend their own ROs to family and friends (Figure 1). Although most scores were positive, some were not. This demonstrates the value in ROs monitoring their profile on Healthgrades, particularly since it is the top search result on Google for patients when searching for individual ROs [6]. 

PRSS were most strongly correlated with trustworthiness, whether physicians spent sufficient time with patients, and ability of the physicians to answer questions and explain conditions (Table 2). PRSS were not as strongly associated with wait times, which contrasts from literature focused on decreasing patient waiting periods in order to improve PRSS [15-17].

Recent evidence across various fields has indicated PRSS has the strongest association with physician-patient relationships [18-20], and our results agree with this finding. A previous study reported that complaints regarding radiology staff member communications were nearly triple to those concerning treatment delays [20]. Other findings show concerns over professionalism were almost twice as frequent as those regarding waiting times [7]. Our study showed strongest correlations between PRSS and trustworthiness and the ability of physicians to answer patient questions, and explain conditions. These were the only factors tested with correlation identified as very strong and conform to results of previous research highlighting the impact ROs’ interpersonal skills may have on PRSS.

The work here highlights the influence of inadequate communication on PRSS. Of the 741 patients in this study who gave ROs less than the highest possible PRSS, the following categories were given scores less than very good at three times the frequency of all other tested variables: trust, whether physicians spent adequate time with patients, and a physician’s ability to answer questions and explain conditions. Many other studies also demonstrate the prominent role of inadequate communication in lower PRSS [18-19]. Previous findings have suggested that confusion amongst patients over method of treatment and choice of equipment has been determined as a primary factor in lower PRSS [21]. Initial attempts at utilizing multimedia to create short presentations to educate patients about radiation treatments have shown promise and warrant continued exploration [22].

Previous studies have looked at the online presence of ROs and determined only 21% of ROs have an online rating on third-party physician rating websites, which is low relative to other specialties [23-24]. This study builds upon previous work by using a much larger sample size of ROs, and our findings indicated that 2679/4444 (60%) of all ROs in the CMS database were found to have a review on Healthgrades. The limited number of reviews may be associated with the lack of online activity by ROs themselves [22]. Our findings highlight a significant difference in LTR scores between physicians with >10 and <10 reviews, underscoring the impact number of reviews can have on physicians ratings. Further steps ROs can take to improve their online identity have been previously discussed [6].

Amidst the rise in online reviews, it is vital that physicians maintain their responsibilities and remember their ethical duties in prevention and treatment of patients’ illness [25]. Furthermore, the limitations of patients’ ability to understand and properly assess all aspects of a physician’s role, especially work completed outside of the patient visit, must always be a consideration. Patients’ lack of medical training and inability to discern much of what is and isn’t under a physician’s control highlight the necessity of not allowing patient perception to completely affect a physician’s style of care. However, in the modern era of technology’s expanding influence, there is much to be gained if ROs focus efforts toward establishing a stronger online presence. Creating and curating appropriate online information for patients may also contribute to greater patient-physician engagement [26-27].

Limitations

This study has limitations. First, sample bias must be acknowledged since we used Healthgrades, a voluntary patient-generated review site. There has been evidence to suggest physicians of lower quality are more likely to be rated online than the average physician while higher quality physicians are less likely to receive online ratings and attention [28]. About 40% of physicians in the database used did not have any physician reviews. Second, the authenticity of patient evaluations and physician self-promotion may be at play. It is possible to leave an anonymous review on Healthgrades for any physician as long as one provides an e-mail address, which is not published on the website. Third, while demographic factors for physicians were analyzed, this information was not available for patients; this precluded analysis of potentially moderating variables in patient satisfaction and geographical location. Fourth, all factors assessed in this survey were assigned quantitative values. While the Healthgrades site provided a 1-5 numerical scale used for most factors, factors such as gender, geography, and number of reviews had to be scaled differently. Many possible numerical scales for these factors can reasonably be used in analysis, and minor variations in values computed can result depending on which scale is chosen.

It is important to highlight that the information in this study is related to subjective ratings, and we are not suggesting that the rating information correlates with an individual RO’s quality. Although the voluntary patient responses are a limitation of the data, it is important to realize that patients may be using these data to potentially make decisions about their physicians. In this way, the ratings hold weight and merit attention even if they are not good measures of overall physician quality. The data presented here illustrates the role of online third-party rating websites in better understanding patient satisfaction in radiation oncology.

## Conclusions

As a whole, satisfaction scores for ROs on Healthgrades, a leading physician ratings website, are very strong. Most patients leaving reviews are likely to recommend their own ROs to their friends and family. Understanding online ratings and identifying factors associated with positive ratings are important for both patients and ROs due to the recent growth in physician-rating third-party sites. ROs should have increased awareness regarding their online reputation and third-party sites like Healthgrades.
